# Salivary analysis of oral cancer biomarkers

**DOI:** 10.1038/sj.bjc.6605290

**Published:** 2009-09-29

**Authors:** T Shpitzer, Y Hamzany, G Bahar, R Feinmesser, D Savulescu, I Borovoi, M Gavish, R M Nagler

**Affiliations:** 1 Department of Otorhinolaryngology, Rabin Medical Center, Petah Tiqva and Sackler Faculty of Medicine, Tel Aviv University, Tel Aviv, Israel; 2 Department of Molecular Pharmacology and Oral Biochemistry Laboratory, Rappaport Faculty of Medicine, Technion – Israel Institute of Technology Haifa, Haifa, Israel; 3 Department of Oral and Maxillofacial Surgical, Rambam Medical Center, Haifa, Israel

**Keywords:** saliva, oral cancer, biomarkers

## Abstract

**Background:**

Oral cancer is a common and lethal malignancy. Direct contact between saliva and the oral cancer lesion makes measurement of tumour markers in saliva an attractive alternative to serum testing.

**Methods:**

We tested 19 tongue cancer patients, measuring the levels of 8 salivary markers related to oxidative stress, DNA repair, carcinogenesis, metastasis and cellular proliferation and death.

**Results:**

Five markers increased in cancer patients by 39–246%: carbonyls, lactate dehydrogenase, metalloproteinase-9 (MMP-9), Ki67 and Cyclin D1 (CycD1) (*P*⩽0.01). Three markers decreased by 16–29%: 8-oxoguanine DNA glycosylase, phosphorylated-Src and mammary serine protease inhibitor (Maspin) (*P*⩽0.01). Increase in salivary carbonyls was profound (by 246%, *P*=0.012); alterations in CycD1 (87% increase, *P*=0.000006) and Maspin (29% decrease, *P*=0.007) were especially significant. Sensitivity values of these eight analysed markers ranged from 58% to 100%; specificity values ranged from 42% to 100%. Both values were especially high for the CycD1 and Maspin markers, 100% for each value of each marker. These were also high for carbonyls, 90% and 80%, respectively, and for MMP-9, 100% and 79%, respectively.

**Conclusion:**

The significance of each salivary alteration is discussed. As all alterations correlated with each other, they may belong to a single carcinogenetic network. Cancer-related changes in salivary tumour markers may be used as a diagnostic tool for diagnosis, prognosis and post-operative monitoring.

Salivary testing, a non-invasive alternative to serum testing, can be an effective modality for diagnosis and prognosis predicting of oral cancer as well as for monitoring the patient's post-therapy status (Nagler *et al*, 2006). Oral cancer (oral squamous cell carcinoma, OSCC) is the sixth most common human malignancy, with a 5-year mortality rate of approximately 50% (Myers *et al*, 2000; Kantola *et al*, 2000), which has not changed significantly in more than 50 years, and a high rate of morbidity (Yuen *et al*, 1998; Ribeiro *et al*, 2000; Sparano *et al*, 2004). The therapeutic modality currently offered to OSCC patients is based on traditional stage-predicting indices (based mostly on the TNM criteria) and on histological grading. Unfortunately, these predictors are subjective and relatively unreliable, as two tumours with identical staging and grading often behave very differently; though one responds to therapy, the other may be lethal. Thus, there has been an ever-growing effort dedicated to the basic research of oral cancer, focusing on the identification of biological indicators for the diagnosis of its biological nature and aggressiveness. However, very few studies have examined tumour markers in the saliva of OSCC patients, though such an examination might be of great benefit because of the direct contact between the oral cancer lesion and saliva. The purpose of this study was to concurrently examine in the saliva of the OSCC (tongue) patients the following eight biomarkers that have been suggested earlier to be related to OSCC (mostly by tissue analysis): carbonyls, 8-oxoguanine DNA glycosylase (OGG1), mammary serine protease inhibitor (Maspin), Ki67, phosphorylated-Src (phospho-Src), Cyclin D1 (CycD1), metalloproteinase-9 (MMP-9) and lactate dehydrogenase (LDH). MMP-9 and LDH have been measured quativavely in saliva of OSCC cancer patients whereas salivary carbonyls were studied by a western gel only. Studies of the other five markers have never been published in the professional literature (Bahar *et al*, 2007; Shpitzer *et al*, 2007). Furthermore, all eight markers have never been studied simultanously, nor have they been related to each other or evaluated for their diagnostic sensitivity and specificity values, or for their mutual pathogenetic role in OSCC.

## Materials and methods

### Patients and study design

The data analysed in this study relate to 19 consecutive patients who were diagnosed with tongue cancer. This study group included 12 females and 7 males, mean age 66±4 (range 27–86), who were compared with a control group with a similar age and gender distribution. The data obtained included staging (according to the TNM criteria), histological grading, depth of the tumour, maximal tumour diameter, localisation at the base *vs* mobile part of the tongue and the patients, smoking habit, age and gender. Analysis of salivary levels of carbonyls, OGG1, Maspin, phospho-Src, CycD1, Ki67, MMP-9 and LDH was performed. These were measured in saliva, which was collected as described earlier (Nagler *et al*, 2002), shortly before the administration of the definitive curative treatment. This included surgical removal of the primary tongue tumour, neck dissection and, in most cases, post-operative adjuvant radiotherapy.

### Immunoreactivity assay for salivary markers

Saliva samples were centrifuged (800 **g**, 10 min, 4°C), and the pellets were suspended in 150 *μ*l of lysis buffer (45 mM HEPES, 0.4 M KCl, 1 mM EDTA, 10% glycerol, pH 7.8). After 30 min incubation at room temperature the samples were centrifuged (11 000 **g**, 10 min, 4°C). Protein concentrations in the supernatants were determined. A volume containing 50 ng of protein was transferred to a 1.5 ml vial and all samples were brought to the same volume of 500 *μ*l with the addition of PBS. The solutions were mixed well and 100 *μ*l of each sample was added to ELISA-plate wells (nunc-immunoplate; Thermo Fisher Scientific, Waltham, MA, USA). The plate was covered and stored overnight at 4°C. The next day, each well was washed three times with 100 *μ*l PBS–Tween solution (PBS-T, PBS containing 0.05% Tween 20) and a volume of 100 *μ*l of 1% BSA PBS-T blocking solution (PBS containing 0.05% Tween 20 and 1% BSA) was added to each well. After 1 h incubation at room temperature, 100 *μ*l of primary antibody was added to each well. After 2 h incubation at room temperature, the plate was washed as described above and a volume of 100 *μ*l of secondary antibody was added to each well. After 2 h incubation at room temperature the plate was washed as described above. To achieve colour development, we added 100 *μ*l of 3,3′,5,5′-tetramethylbenzidine solution (Southern Biotech, Birmingham, AL, USA) to each well. After 1–2 min, we added 100 *μ*l of stopping reagent to each well (10% sulphuric acid). Absorbencies of the samples, representing the levels of the specific proteins examined, were measured at the wavelength 450 nm directly after the addition of the stopping reagent, using a Zenith 200 ELISA reader (Anthos, Eugendorf, Austria). For MMP-9, we used a polyclonal rabbit anti-human antibody (1 : 1000; Sigma-Aldrich, Saint Louis, MO, USA). For OGG1, we used a polyclonal rabbit anti-human antibody (1 : 10000; Alpha Diagnostic International, San Antonio, TX, USA). For phospho-Src, we used a polyclonal rabbit anti-human antibody (1 : 1000; Sigma-Aldrich). For Ki67, we used a monoclonal rabbit anti-human antibody (1 : 1000; Acris Antibodies, Herford, Germany). For Maspin, we used a polyclonal rabbit anti-human antibody (1 : 1000; Sigma-Aldrich). For CycD1, we used a polyclonal rabbit anti-human antibody (1 : 500; Sigma-Aldrich). For all assays we used a peroxidase-conjugated goat anti-rabbit secondary antibody (1 : 5000; Jackson Immunoresearch, West Grove, PA, USA).

### Detection of protein oxidation (protein carbonyl assay)

An enzyme-linked immunosorbent assay (ELISA) colorimetric test kit (BioCell Corporation Ltd., Papatoetoe, New Zealand) was used to quantitatively measure the products of protein oxidation (carbonyls) in saliva samples. Samples were centrifuged (800 g, 10 min, 4°C), and the pellets were suspended in 150 *μ*l of lysis buffer (45 mM HEPES, 0.4 M KCl, 1 mM EDTA, 10% glycerol, pH 7.8). After 30 min incubation at room temperature the samples were centrifuged (11 000 **g**, 10 min, 4°C) and the supernatants were stored at −20°C. On the day of the carbonyl analysis, the supernatants were thawed and protein concentrations were determined. A volume of 20 *μ*g was transferred to a 1.5 ml vial and all samples were brought to the same volume of 100 *μ*l with the addition of water of high-pressure liquid chromatography grade. We added 0.8 volumes of ice cold 28% trichloroacetic acid, mixed well, and after 10 min of incubation on ice the tubes were centrifuged (10 000 g, 3 min, 4°C). Supernatants were carefully aspirated without disturbing the pellet; 5 *μ*l of EIA buffer (1 M phosphate solution containing 1% BSA, 4 M NaCl, 10 mM EDTA and 0.1% sodium azide) and 15 *μ*l diluted 2,4-dinitrophenol (DNP) solution were added to pellets according to the manufacturer's instructions. After 45 min incubation at room temperature, 5 *μ*l of each sample was taken to a parallel set of 1.5 ml vials containing 1 ml EIA buffer. The solutions were mixed well and 200 *μ*l of each sample was added to ELISA-plate wells. The plate was covered and stored overnight at 4°C. The next day, the plate was washed three times with EIA buffer (250 *μ*l per well) and 250 *μ*l of diluted blocking solution (provided by the manufacturer) was added to each well. After 30 min incubation at room temperature, the wells were washed as described above and 200 *μ*l of diluted anti-DNP-biotin-antibody was added to each well. The plate was incubated for 1 h at 37°C. After incubation, the plate was washed and 200 *μ*l of diluted streptavidin–HRP was added to each well. After 1 h incubation at room temperature the plate was washed as described above. To achieve colour development, we added 200 *μ*l of chromatin reagent (provided by the manufacturer) to each well. After 5 min, we added 100 *μ*l of stopping reagent to each well. Absorbencies of the samples were measured at the wavelength 450 nm directly after the addition of the stopping reagent, using a Zenith 200 ELISA reader (Anthos). To quantify the absorbance values, we performed the same procedure for standard and control samples provided by the manufacturer, and created a standard curve.

### LDH activity

For the measurement of LDH activity, saliva samples were diluted by a factor of 10 using double-distilled water. The activity of LDH was determined by kinetic spectrophotometry using a commercial kit (REF DF53A, Siemens Healthcare Diagnostics, Deerfield, IL, USA) and a Dimension RXL analyser (Siemens Healthcare Diagnostics).

### Statistical analysis

Data concerning the levels of various markers were evaluated in saliva, and the mean, standard deviation (s.d.) and mean standard error (s.e.) values were analysed and compared with the two-sample *t*-test for differences in means. The criterion for statistical significance was *P*<0.05. The correlations between the marker levels in saliva were analysed using the Pearson correlation analysis. A correlation matrix of estimators was used to analyse the correlation coefficients between the salivary parameters. For classification analysis, cutoff values were calculated as mean plus/minus 1 s.d. value of healthy controls. Sensitivity and specificity values were calculated as the fraction of observations, which were correctly classified.

## Results

### Clinical data staging, pathological grading, dimensions, site and extension of the tumours

The distribution of the 19 patients according to tumour size (T) showed that 7 had T1 and 8 patients had T2 tumours whereas only 2 patients had T3 and 2 patients had T4 tumours. That is, nearly 80% of the patients had early (small to moderate) tumours. In 13 out of 19 (68%) of the patients there were no neck metastases (N0) whereas 4 patients were diagnosed with N1 and 2 with N2. None had distant metastasis (all patients were M0). Accordingly, 68% of the patients were diagnosed with early stage tumours (1+2) whereas only 32% were diagnosed with advanced stages (3+4). Similarly, most of the patients (84%) were diagnosed with well differentiated and moderately differentiated tumours and only three patients were diagnosed with poorly differentiated lesions. In 16% of the patients (3 out of 19) the tumour extended beyond the lingual region and expanded locally towards neighbouring regions, towards the floor of the mouth.

The mean tumour diameter at diagnosis was 3.4±0.9 cm (range 0.5–8.0 cm) and the mean depth was 3.4±0.9 mm (range 1–25 mm). Only 12.5% of the patients smoked (2 of the 16 for whom this information was available). The rate of smokers in the control group was not significantly different. Only 2 out of 19 patients had a previous pre-malignant lesion (Lichen planus), only 1 out of 17 patients (for whom the data were available) had other previous malignancy and none had been treated earlier with radiotherapy. None of the controls was treated with radiotherapy or had head and neck cancer earlier.

### Salivary tumour markers

**Figure 1:**
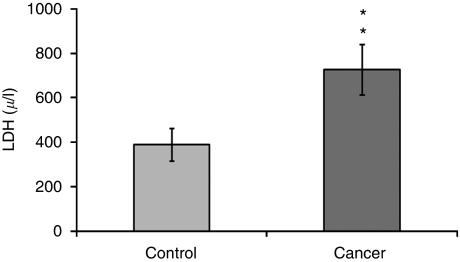
Mean activity of salivary LDH (*μ*/l) in healthy controls (Control, *n*=19) and oral cancer patients (Cancer, *n*=19), (^**^ *P*=0.002).

Salivary tumour marker analysis showed highly significantly changes in the levels of all eight markers analysed (Table 1; Figure 1). Five of these were increased in the cancer patients by 39–246%: carbonyls, Ki67, CycD1, MMP-9 and LDH (*P*⩽0.01). The other three markers were decreased in the cancer patients by 16–29%: OGG1, Maspin and phospho-Src (*P*⩽0.01).

The salivary mean (±s.e.) concentrations (OD values) in controls of MMP-9, carbonyls, Ki67, CycD1, OGG1, phospho-Src and Maspin were 0.04±0, 0.37±0.23, 0.15±0.05, 0.70±0.03, 0.50±0.02, 0.67±0.03 and 0.44±0.02, respectively. The increase in salivary carbonyls was profound (by 246%, *P*=0.012) and especially significant were the alterations in CycD1 (an increase by 87%, *P*=0.000006) and in Maspin (a decrease by 29%, *P*=0.007) (Table 1). The salivary mean (±s.e.) LDH activity value (the only value presented absolutely) in controls was 390±73 *μ*/l (Figure 1). The sensitivity values of the eight analysed markers were in the range of 58–100% whereas the specificity values were in the range of 42–100% (Table 1).

The sensitivity and specificity values were especially high for the CycD1 and Maspin markers, 100% for each value of each marker. These were also quite high for the carbonyls, 90% and 80%, respectively, and for the MMP-9, 100% and 79%, respectively.

Multiple significant (*r*<−0.4 or >0.4) correlations were shown among all eight markers, each with some of the others. The most significant correlations were shown between: Maspin and CycD1 (0.89), carbonyls and CycD1 (0.79), carbonyls and Maspin (0.75) and carbonyls and Ki67 (0.72). In addition, quite high were the significant correlations between CycD1 and MMP-9 (0.67) and between Maspin and OGG1 (0.62) (Table 2).

## Discussion

A most important result found in this study is that all eight salivary parameters analysed in the cancer patients were altered in a highly significant manner, and were characterised by relatively high sensitivity and specificity values. Moreover, all markers ‘talked with each other’, that is these alterations significantly correlated among themselves, indicating that they all belong to a single carcinogenetic network that, when fully understood, may be used for the development of anti-cancer drugs related to OSCC. We thus believe that the demonstrated results are of a significant merit with respect to both the clinical and the pathogenesis-related aspects of oral cancer. These significant demonstrated alterations in salivary markers of the cancer patients may be used as a diagnostic tool, especially when a concurrent analysis is performed for several salivary markers. Furthermore, this diagnostic tool is of special importance for patient monitoring, as it is often very difficult to distinguish clinically between a post operative and/or irradiated scarred oral mucosa and a recurring cancer lesion. Accordingly, such an analysis might turn into a valuable diagnostic tool and it might save many unnecessary biopsies and hospital/out patient clinic visits. Carbonylation (indication of oxidative damage to proteins) has attracted a great deal of attention in cancer research because of its irreversible and unrepairable nature, becoming cytotoxic and associated with cancer (Nystrom, 2005). The currently reported substantial increase in salivary carbonyls (by 246%) in the OSCC patients is of no surprise, pointing at the significant free radicals attack to which the epithelial cells have been exposed. Similarly, it was recently reported that in malignant tissues (in transitional meningioma and in glioblastoma multiforme) the degree of oxidative DNA damage (8OHdG) is increased whereas the total anti-oxidant capacity is decreased (Hanimoglu *et al*, 2007; Tuzgen *et al*, 2007) Indeed, efficient DNA repair mechanisms comprise a critical component in the protection against cancer and among these the 8-oxoguanine DNA glycosylase (OGG1) enzyme is crucial for repairing the oxidative DNA lesion 8OHdG that is highly mutagenic and carcinogenic. Most importantly is that reduced activity of OGG1 is considered an established risk factor for various cancers such as lung and head and neck cancer (Paz-Elizur *et al*, 2006, 2008). Hence, the reduction observed in salivary OGG1 in the OSCC patients is expected. In a similar manner, the reduction we found for Maspin is expected. This as Maspin is a tumour supressor protein that was shown to suppress tumour growth and progression, angiogenesis, invasion and metastasis in various malignancies including head and neck cancer (Cho *et al*, 2007; Iezzi *et al*, 2007; Marioni *et al*, 2008). Accordingly, its reduction is expected to promote carcinogenesis. In addition, the reduction we noted for phospho-Src can be explained. Phospho-Src is the inhibited form of Src and though the latter is expected to be increased, the first is expected to be decreased in cancer patients as indeed we noted. A major function of Src (a cytoplasmic kinase) is to drive adhesion changes that are associated with transition, proliferation and metastasis (Avizienyte *et al*, 2005; Chen *et al*, 2008). Reversible phosphorylation of Src by oxidants and other agents turn it into its inhibited form, the phospho-Src. In contrast to OGG1, Maspin and phospho-Src, which were reduced, we found an increase in the levels of the salivary CysD1, Ki67, LDH and MMP-9 in the OSCC patients. CycD1 and Ki67 are cell-cycle regulators, which have been shown to be correlated with cellular proliferation and tumour progression, metastasis and poor prognosis (Liu *et al*, 2003; Adjei, 2005; Wang *et al*, 2006) and accordingly are expected to increase in tumours. LDH was found to increase in the serum of various malignancies and has been identified as the main recurrent adverse prognostic factors (Schneider, 2006; Duffy and Crown, 2008; Culine, 2009). As for the demonstrated increase in MMP-9, it is worth noting that it was shown earlier to be elevated in saliva (Shpitzer *et al*, 2007) and that strong stromal MMP-9-staining intensity was correlated with poor tumour differentiation (Kosunen *et al*, 2007). MMP-9 are metalloproteases that have been shown to participate in cancer pathogenesis as they degrade type IV collagen, a major component of basement membrane, as well as other types of collagens (V, VII and X), elastin and fibronectin. They are highly expressed in stromal cells surrounding the invading front of metastasising tumours and their levels are elevated in tumour endothelium and in urine of cancer patients (Pories *et al*, 2008; Smith *et al*, 2008; Chen *et al*, 2009). Moreover, MMP-9 polymorphism was shown to have a strong association with increased risk for developing OSCC whereas constitutive expression and secretion of MMP-9 in invasive OSCC cell lines were shown as well (Vairaktaris *et al*, 2008).

In summary, the highly significant changes showed for all eight biomarkers analysed in OSCC patients are encouraging in light of the many advantages of saliva measurement. It would be highly desirable and beneficial if salivary tumour marker analysis could be performed on a routine basis as salivary harvesting is non-invasive, being an effective alternative to serum testing, and the possibility of developing self, home testing kits for such markers further facilitates it as a diagnostic aid. That is especially important for people who live far from treatment centres and especially for those at high risk for developing oral cancer (such as patients with previous OSCC or with pre-malignant lesions). Furthermore, this study sheds further light on the role of the analysed tumour-related proteins (markers) in the OSCC pathogenetic network and also points at a unique opportunity that we may have to intervene with local therapeutic agents that can be easily applied to the oral mucosa.

## Figures and Tables

**Table 1:** Statistical analysis of the eight analysed salivary biomarkers

**Parameter**	**% Of change (out of control)**	***P***	**Sensitivity (%)**	**Specificity (%)**
MMP-9	39	0.014	100	79
Carbonyls	246	0.012	90	80
OGG1	−16	0.007	77	75
phospho-Src	−24	0.010	77	75
Ki67	127	0.015	58	67
Maspin	−29	0.001	100	100
LDH	86	0.002	79	42
CycD1	87	<0.00001	100	100

All were found to be highly significantly altered in the saliva of oral cancer patients as compared with controls. The following were calculated: percentage of change in the mean levels of each parameter, statistical significance of the change (represented by *P*), sensitivity and specificity values for each biomarker. MMP-9=metalloproteinase-9; OGG1=8-oxoguanine DNA glycosylase; phospho-Src=phosphorylated-Src; Maspin=mammary serine protease inhibitor; LDH=lactate dehydrogenase; CycD1=Cyclin D1.

**Table 2:** List of biomarkers that were found to be significantly correlated (*r* – Pearson correlation coefficient; *r*<−0.4 or >0.4 – significant correlation)

**Parameters compared**	***r***
MMP9–CycD1	0.67
MMP9–carbonyls	0.57
MMP9–Ki67	0.48
Carbonyls–OGG1	−0.57
Carbonyls–Ki67	0.72
Carbonyls–Maspin	−0.75
Carbonyls–CycD1	0.79
Carbonyls–LDH	0.56
OGG1–Src	0.55
OGG1–Maspin	0.62
OGG1–CycD1	−0.54
OGG1–LDH	0.42
Ki67–CycD1	0.54
Maspin–CycD1	−0.89

MMP-9=metalloproteinase-9; OGG1=8-oxoguanine DNA glycosylase; phospho-Src=phosphorylated-Src; Maspin=mammary serine protease inhibitor; LDH=lactate dehydrogenase; CycD1=Cyclin D1.
